# Machine Learning Methods for the Prediction of Intraoperative Hypotension with Biosignal Waveforms

**DOI:** 10.3390/medicina61112039

**Published:** 2025-11-14

**Authors:** Jae-Geum Shim, Wonhyuck Yoon, Sang Jun Lee, Se-Hyun Chang, So-Ra Jung, Jun Young Chung

**Affiliations:** 1 Department of Anesthesiology and Pain Medicine, Kangbuk Samsung Hospital, Sungkyunkwan University School of Medicine, Seoul 03181, Republic of Korea; jgshim77@naver.com; 2 OUaR LaB, Inc., Seoul 03080, Republic of Korea; 3 MISO Info. Tech. Co., Ltd., Seoul 13824, Republic of Korea; 4 Department of Medical Informatics, College of Medicine, the Catholic University of Korea, Seoul 14662, Republic of Korea; 5 Konan Technology Inc., 327 Gangnam-Daero, Seocho-Gu, Seoul 06627, Republic of Korea; 6 Department of Anesthesiology and Pain Medicine, College of Medicine, Kyung Hee University, Seoul 02447, Republic of Korea

**Keywords:** predict, intraoperative hypotension, deep learning, machine learning

## Abstract

*Background and Objectives*: Intraoperative hypotension (IOH) is of great importance in preventing diseases such as postoperative myocardial infarction, acute kidney injury, and mortality. This study aimed to develop and validate machine learning and deep learning models that predict IOH using both biosignals and personalized clinical information for each patient. *Materials and Methods*: In this retrospective observational study, we used the VitalDB open dataset, which included intraoperative biosignals and clinical information from 6388 patients who underwent non-cardiac surgery between June 2016 and August 2017 at Seoul National University Hospital, Seoul, South Korea. The predictive performances of models trained with four waveforms (arterial blood pressure, electrocardiography, photoplethysmography, and capnography) and clinical information were evaluated and compared at time points at 5 min before the hypotensive event. To predict hypotensive events during surgery, we developed two predictive models: machine learning and deep learning. In total, 2611 patients were enrolled in this retrospective study. Machine and deep learning algorithms were developed and validated using raw waveforms and clinical information as inputs. *Results*: Gradient boosting machine showed predicted IOH with an AUROC and accuracy of 0.94 (0.93–0.95) and 0.88 (0.86–0.89). A hybrid CNN-RNN model also showed similar performance with an AUROC and accuracy of 0.94 (0.93–0.95) and 0.88 (0.87–0.89). *Conclusions*: This study developed and validated machine and deep learning models to predict IOH using waveform data and covariate values. In the future, we anticipate that the results of our study will contribute to predicting IOH in real time in the operating room and reducing the occurrence of IOH.

## 1. Introduction

Intraoperative hypotension (IOH) is of great importance for preventing diseases such as postoperative myocardial infarction, acute kidney injury, and mortality [1,2,3]. Researchers have found that hemodynamic instability due to low blood pressure is associated with negative clinical outcomes in high-risk patients [4,5]. Therefore, maintaining proper blood pressure during surgery and anesthesia could prevent poor postoperative outcomes. However, there is no consensus regarding the ideal blood pressure to adequately maintain organ perfusion [6]. During surgery, the anesthesiologist continuously analyzes many biosignals from the monitor and takes appropriate measures according to the patient’s high or low blood pressure. Nevertheless, predicting hypotensive events in advance by observing the various hemodynamic parameters generated by monitoring devices is challenging.

Electrocardiography (ECGs) and cardiac monitoring are readily available and commonly used in operating rooms. Arterial blood pressure (ABP) is also related to electrocardiographic changes, which have been observed in cardiac rhythm, ischemia, and electrolytes in a few studies [7,8]. In recent years, the estimation of blood pressure using photoplethysmography (PPG) has gained considerable attention. PPG-only based methods extract morphological features of blood volume dynamics derived from PPG waveforms at a particular site [9]. Moreover, use of combined photoplethysmography and electrocardiogram signals extracts the intrinsic waveform information related to blood volume propagation through arteries [10,11]. End-tidal carbon dioxide (ETCO_2_) is the level of carbon dioxide in exhaled air measured at the end of expiration. In a previous study, cardiac output and lung perfusion were shown to affect ETCO_2_ [12]. ETCO_2_ could also represent the effectiveness of cardiopulmonary resuscitation in terms of circulating blood flow [13]. Therefore, the rapid drop in ABP may be related to changes in ETCO_2_.

The Hypotension Prediction Index (HPI) is a commercial system based on a high-fidelity arterial waveform by Edwards Lifesciences (Irvine, CA, USA) [14]. The HPI uses 23 features of the arterial waveform with the best performance selected from a possible combination of more than 2.6 million features for the logistic regression model. This machine learning model predicts IOH up to 15 min before its occurrence, using several parameters, including stroke volume estimation. Whether HPI are effective in reducing the frequency and duration of hypotensive events in clinical settings is controversial. Some studies demonstrated that the HPI achieved statistically significant reduction in hypotension compared to standard of care [15]. However, intraoperative HPI-guided hemodynamic management did not reduce postoperative hypotension [16].

As biosignals, including waveforms and numeric data, are saved and processed in real time, deep learning technologies have been widely applied to manage time-series data in the operating room. In previous studies, 1-dimensional convolution neural networks (CNN) were implemented to predict ABP with only four waveforms of data, and reasonable results were derived [17,18]. In addition, recurrent neural networks (RNN) for handling biosignals in the form of sequential data have shown adequate predictive performance for ICU readmission and in-hospital cardiac arrest [19,20].

Most existing studies on predicting IOH using machine learning are limited because they rely only on waveform biosignals, including arterial pressure and PPG waveforms. However, preoperative risk factors may also help screen patients who are hemodynamically unstable and thus prone to hypotension during surgery. We anticipated that combining multiple waveforms with patient clinical information would better predict intraoperative hypotension. This study aimed to develop and validate machine learning and deep learning models that predict IOH using both biosignal and clinical information personalized for each patient.

## 2. Methods

### 2.1. Data Source and Study Approval

Data were extracted from VitalDB, a public data repository of biosignal waveforms and clinical information from June 2016 to August 2017 at Seoul National University Hospital, Seoul, South Korea. A waiver of study approval was granted by the Institutional Review Board (IRB) of Kangbuk Samsung Hospital due to the retrospective design and use of de-identified data (IRB No. KBSMC 2023-02-002).

### 2.2. Participants

The dataset was obtained from 6388 patients who underwent non-cardiac surgery in 10 operating rooms. Data were recorded using VitalRecorder, an automatic data recording software. Biosignals including arterial pressure, ECG (lead II), PPG, and ETCO_2_ waveform as well as clinical information were used to improve the performance in our predictive model [21]. The exclusion criteria were as follows: (1) pediatrics (age < 18); (2) the patients for whom at least one of all four waveforms were not available; (3) any missing values for preoperative clinical variables including age, sex, BMI, ASA physical status, emergency surgery, and hypertension; and (4) any missing values for preoperative laboratory data including WBC count, hemoglobin, BUN, Cr, albumin, Na, K.

### 2.3. Data Preparation

We collected data on biosignal waveforms, and preoperative clinical and laboratory variables. The biosignal waveforms included arterial pressure, ECG (lead II), PPG, and ETCO_2_. The preoperative clinical variables included age, sex, body mass index (BMI), American Society of Anesthesiologists (ASA) physical status, emergency surgery, and hypertension. Preoperative laboratory variables included white blood cell (WBC) count, hemoglobin, blood urea nitrogen (BUN), creatinine, albumin, sodium (Na), and potassium (K).

ABP, ECG, PPG, and ETCO_2_ waveforms were collected at an acquisition interval of 1/500 (s). All four sets of waveform data were resampled at 100 Hz. Peak detection algorithms implemented in Python 3.8.10 (https://github.com/scipy/) (accessed on 1 March 2024) were used to extract all ABP peaks from the arterial pressure waveform and identify each cardiac cycle.

To remove artifacts in the arterial pressure waveforms, we excluded any segment in which the cardiac cycle length was too long or small beyond the normal physiological range, as well as segments with a mean arterial pressure (MAP of <20 or >200 mmHg).

**Figure 1:**
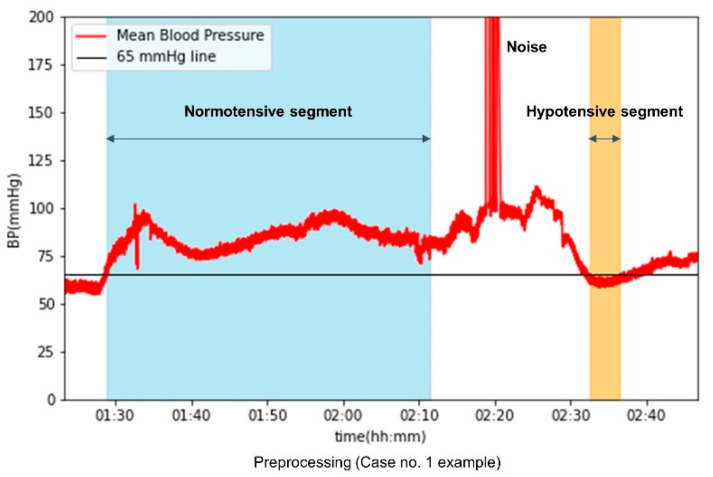
Example of data preprocessing.

In our study, we defined hypotensive events as any rhythm segment where hypotension lasted for more than 1 min; that is, MAP was less than or equal to 65 mmHg, regardless of the length of the interval (Figure 1). To predict hypotensive events, the input segments of 30 s ABP, ECG, PPG, and ETCO_2_ waveforms were obtained 5 min before each event. To sample non-hypotensive events, rhythm segments for more than 20 min in which the MAP was maintained above 65 mmHg were extracted. The 30 s input segments were obtained for a 5 min prediction of non-hypotensive events. For the predictive model development, one or two inputs were extracted from the non-hypotensive segment to balance the two data events.

### 2.4. Model Building

In this study, we developed two models to predict hypotensive events during surgery: machine learning and deep learning. Our proposed model is a binary classifier that predicts one of two classes, namely a hypotensive event or a non-hypotensive event five minutes before the event occurs, by analyzing the four biosignal waveforms and clinical information. This study implemented two models based on machine learning, namely, a gradient boosting machine (GBM) and a hybrid model of CNN with RNN, known as a hybrid CNN-RNN, to assess the accuracy of predicting intraoperative hypotension.

### 2.5. Machine Learning Model: Gradient Boosting

**Figure 2:**
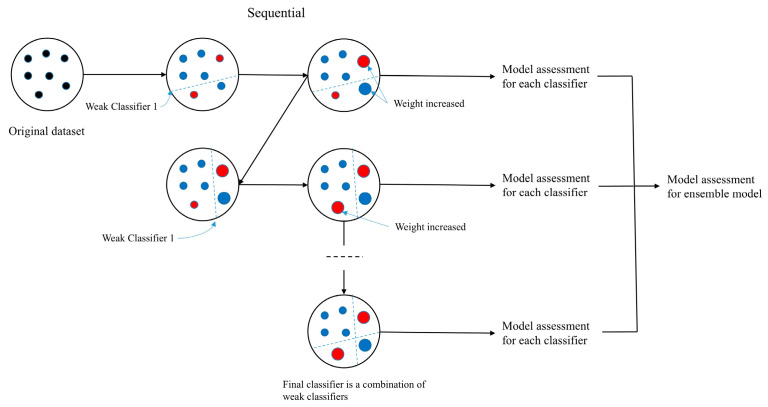
Flow diagram of gradient boosting machine algorithm.

Boosting is an ensemble learning technique in which the predictors are generated sequentially. This method transforms weak learners into strong ones. Boosting begins with the concept that subsequent classifiers learn from the mistakes of previous classifiers. Gradient boosting is a machine learning algorithm to solve statistical regression as well as classification problem [22]. Its concept is based on ensemble learning, which generates a prediction model by assembling weak prediction models, generally decision trees [23,24]. In other words, it combines all weak models, and after the combination of various weak models, forms a stronger predictive model to improve the accuracy (Figure 2).

Gradient boosting proceeds in the following stages: First, a base model was constructed. After analyzing the first base model, the weights of all observations that failed to be classified correctly were increased. However, this reduces the weight of observations that do not have correctly classified problems. Therefore, the new model used was a combination of the first and second classifiers. The algorithm then computed the prediction error from the ensemble model and constructed a final classifier to predict the amended residuals. In total, the above process is repeated for a certain number of iterations.

For the gradient boosting model, hand-crafted features were extracted from a 30 s peri-event window preceding the prediction point. Each biosignal (ABP, ECG, PPG, and ETCO_2_) was segmented into ten equal non-overlapping sub-windows (3 s each), and summary statistics were computed to represent both the absolute physiological state and short-term dynamic changes. Specifically, the mean value of each sub-window was used as the primary input feature, capturing temporal evolution and signal trends across the observation period. In addition, preoperative clinical and laboratory variables were incorporated to reflect baseline physiological status. These engineered features enabled the gradient boosting model to learn physiologic patterns without direct access to raw time-series data, serving as a structured representation parallel to the deep learning model that processed continuous waveform inputs.

### 2.6. Deep Learning Model

**Figure 3:**
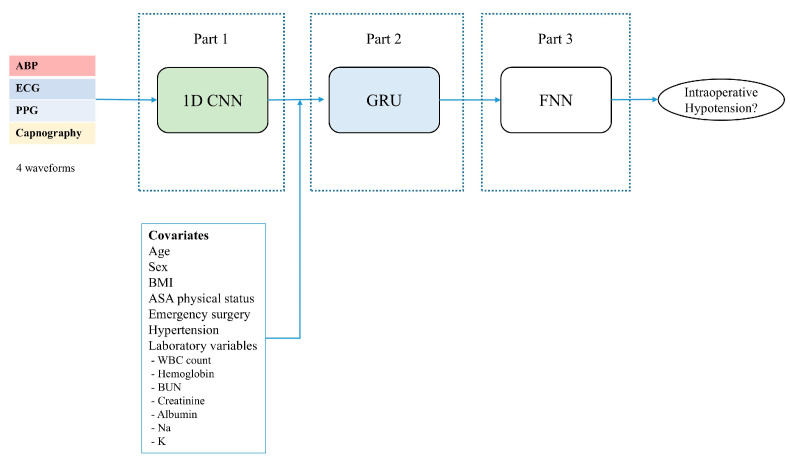
Schematic of hybrid CNN-RNN model.

The model architecture of the deep learning method is shown in Figure 3. In deep learning, each level is trained to transform the input data into an abstract and complex representation. The deep learning model in our study aims to present the structure of combining a CNN with an RNN to predict IOH based on the patient’s clinical information and vital signs 5 min prior. CNN are effective for extracting features from input data and have been shown to be very good at identifying patterns that are difficult to find using traditional methods. In this study, we developed a model based on hybrid CNN-RNN deep learning consisting of three stages. First, a CNN is used to extract the feature map of the four waveform signals, and then a gated recurrent unit (GRU) is implemented to learn temporal features. The last part is a fully connected network that provides binary class prediction.

### 2.7. Statistical Analysis

Descriptive characteristics of the study population were analyzed using VitalDB data. Continuous variables are presented as mean ± standard deviation. Categorical variables are presented as absolute (*n*) and percentage (%). The performance of the binary classification models in predicting IOH was measured using the area under the receiver operating characteristic (AUROC) curve. We also calculated accuracy, sensitivity, and specificity. Python 3.8.10 (Python Software Foundation, Wilmington, DE, USA) was used to pre-process the signal and develop and validate the model. R version 4.2.1. (R Development Core Team, Vienna, Austria) was used for data visualization and statistical analysis. Statistical significance was set at *p* < 0.05.

### 2.8. Model Assessment

To screen for a disease, particularly to detect IOH in our study, we must measure the validity of that test.

The confusion matrix is a performance measure for the machine learning classification problem, where the output can be two classes. It can be used to estimate the relationship between actual and predicted values using four different combinations. For a given classification test, the performance of the predictive model was measured based on its attributes. Sensitivity, specificity, accuracy, and AUROC are commonly used statistics to quantify the diagnostic ability of the test.

**Figure 4:**
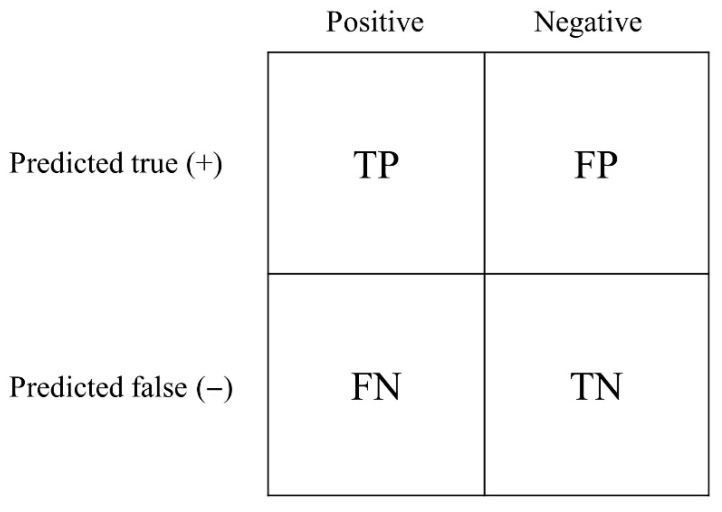
Confusion matrix for binary classification.

Figure 4 shows a table of confusion matrices. True positive (TP) suggests that the person has the disease, and the test result is positive. True negative (TN) indicates that the person does not have the disease, and the test result is negative. Both false positive (FP) and false negative (FN) results indicate that the test results are inconsistent with the actual condition.

Sensitivity is the ability of a model to correctly classify patients with a disease and is defined as

$$Sensitivity=\frac{TP}{TP+FN}$$

A sensitivity test helps rule out a disease for people who test negative.

Specificity is the ability of a model to correctly classify people without disease and is defined as.

$$Specificity=\frac{TN}{TN+FP}$$

A specificity test helps rule a disease for people who test positive.

The accuracy was defined as the proportion of true results (both true-positive and true-negative) in the total number of selected populations.

The accuracy of the model is defined as follows:

$$Accuracy=\frac{TP+TN}{TN+TP+FN+FP}$$

The receiver operating characteristic (ROC) curve shows the relationship between sensitivity and specificity. The AUROC is a performance measurement of a classification model at various threshold settings for diagnostic testing. This performance metric can be used to evaluate binary and multiclass classification models.

### 2.9. Data Availability

The data analyzed for the development and validation of the models in this study are available in VitalDB (https://vitaldb.net, accessed on 1 March 2023).

## 3. Results

### 3.1. Participants and Dataset

**Figure 5:**
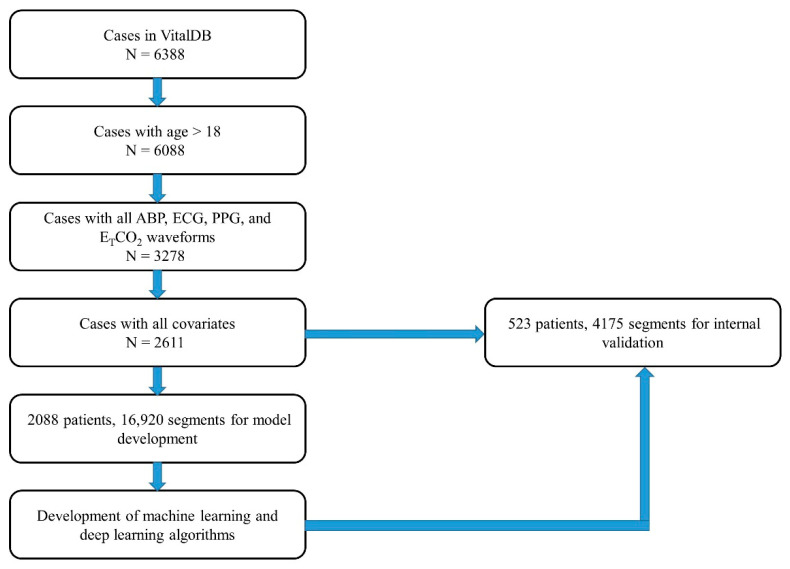
CONSORT diagram in data acquisition.

The total VitalDB dataset consisted of 6388 patients, and 6088 patients were eligible after applying age above 18 years, height and weight range restrictions, and the exclusion of transplantation anesthesia cases. Among them, 3278 patients including all four waveform datasets for ABP, ECG, PPG, and ETCO_2_ were available for the study. Finally, a total of 2611 patients were enrolled in this retrospective study for analysis because of missing values in covariates for preoperative clinical variables, including age, sex, BMI, ASA physical status, emergency surgery, hypertension, and preoperative laboratory data, including WBC count, hemoglobin, BUN, Cr, albumin, Na, and K. These were divided into 2088 patients (16,920 segments) for model development and 523 patients (4175 segments) for internal validation. No overlap occurred between training and validation cohorts. A CONSORT-style flow diagram (Figure 5) details counts and reasons at each step.

The demographic and clinical characteristics of the 2611 patients are summarized in Table 1. No significant differences were observed between the training and test datasets. The mean age of the total dataset was 59.8 (SD 14.0) years. Male patients comprised 55.6% (*N* = 1451) of the patients.

### 3.2. Model Performance

**Figure 6:**
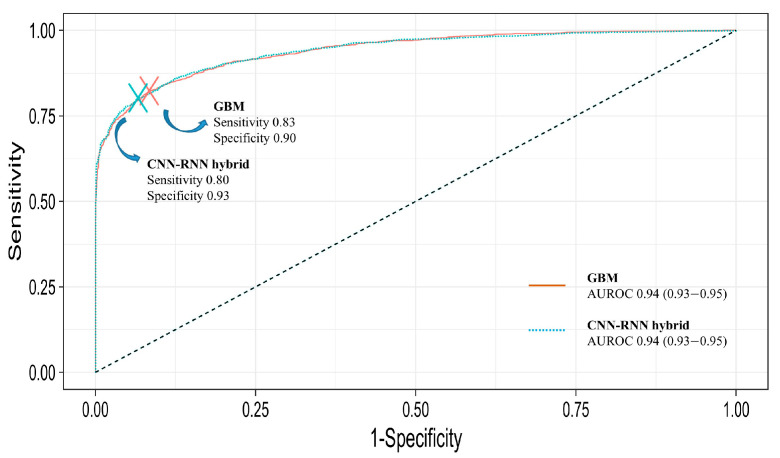
Receiver operating characteristic curves for GBM and hybrid CNN-RNN model.

The prediction performance was presented as the AUROC, accuracy, sensitivity, and specificity 5 min before the event. Table 2 shows the hypotension prediction performance of the classification models with respect to the AUROC. The area under the AUROC was 0.94 for both the GBM and hybrid CNN-RNN models. The AUROC was plotted to show both classification models. Figure 6 summarizes the ROC curves of the two models. Other metrics with the optimal threshold maximizing the sum of the sensitivity and specificity are listed in Table 2. Although the two models demonstrated similar discrimination (AUC 0.94 vs. 0.94, DeLong *p* < 0.001), the error profiles differed. The GBM model showed higher sensitivity (0.83 vs. 0.80, *p* < 0.001) whereas the CNN-RNN demonstrated higher specificity (0.93 vs. 0.90, *p* < 0.001). These results suggest a clinically relevant operating-point trade-off between false-negative and false-positive predictions rather than superiority in overall discrimination.

## 4. Discussion

In this retrospective study, we developed machine and deep learning models to predict IOH five minutes before hypotensive events based on patient clinical information and waveform data of ABP, ECG, PPG, and ETCO_2_. The four waveforms used in this study were monitored in all the operating rooms. Both models showed adequate predictive performance for patients undergoing non-cardiac surgery. Compared to previous studies, the addition of patient clinical information to biosignal data improved the model performance in predicting the occurrence of hypotensive events [18].

Unlike prior IOH predictors that typically exploit a single waveform (e.g., arterial pressure) or raw CNNs without explicit clinical context, our framework integrates four biosignals with perioperative clinical and laboratory variables [18]. This multi-modal design allows the model to capture both short-term hemodynamic dynamics and patient-specific baseline risk, and we show that a feature-based GBM and a raw-waveform CNN-RNN achieve similarly high discrimination while offering complementary interpretability.

Various patient characteristics, clinical information, and types of surgery or anesthesia are associated with intraoperative hypotensive events. Male sex, old age, emergency surgery, and a high ASA of Anesthesiologists physical status were known risk factors. However, preoperative information alone is insufficient to reflect the situation during surgery. Recently, studies have been conducted to predict real-time blood pressure using deep learning methods [17,18]. To the best of our knowledge, no study has applied and compared machine learning and deep learning using biosignals and preoperative information for the prediction of hypotensive events. We hope that the results of this study will contribute to the prediction of hypotension in real time and improve the performance of real-time hypotension prediction.

Currently, HPI is commercialized and used in the operating room to predict real-time IOH in relatively sick patients or during major procedures. The HPI was designed to work in conjunction with commercial sensors such as FloTrac and CO-Trek. HPI uses only arterial waveform features; therefore, it requires a good-quality arterial line waveform. However, in actual clinical settings, the quality of the arterial line often becomes poor in the following situations: damped arterial waveforms, changes in patient position, and arterial catheter flushing. Because our proposed models use four waveforms and clinical information simultaneously, we expect them to be less affected by noise for the arterial line compared with the method relying only on the arterial waveform. This study showed a slightly improved performance compared to a previous study in predicting IOH five minutes before hypotensive events using the HPI (AUROC 0.94 vs. 0.92) [25].

The results of this study show that there is no significant difference in the IOH predictive performance of deep learning and gradient boosting models, which is one of the most popular machine learning algorithms. However, it was confirmed that both models performed better than existing logistic regression-based models. In machine learning and deep learning methods using raw waveform data, there is a possibility that fine morphological differences can be detected in waveform data, but not in logistic regression models using features extracted from raw data [26].

In this study, the criterion for IOH was an absolute threshold MAP of 65 mmHg. However, there is currently no critical standard for IOH. Depending on the type of surgery and the individual characteristics of the patient, the optimal blood pressure threshold may differ [27]. Even each practitioner who controls the patient’s hemodynamic status in the operating room may consider various appropriate standards. In this study, we solved this problem using a binary classification model. However, in future studies, it will be possible to predict blood pressure values accurately using a regression model.

This study has several limitations. First, even though the sample size of our study was small (*n* = 2611), external validation was required because our study was based on single-center data. Future research should include a multicenter study to differentiate the characteristics of the patient groups. All patients were monitored by invasive arterial pressure monitoring. However, in actual clinical settings, this is only a part; thus, it is necessary to create a model with data applied using non-invasive arterial pressure monitoring. Moreover, it is essential to validate the model developed in this study through a prospective study to confirm the generalizability to different data and the actual benefits in clinical trials. Finally, it is necessary to develop a web or mobile application that can be used in real time so that anyone can easily access it.

## 5. Conclusions

In conclusion, the authors demonstrated that the use of the four waveforms commonly monitored in the operating room as well as the patient’s preoperative clinical information would result in good performance in predicting IOH. This study developed and validated machine and deep learning models to predict IOH using both waveform data and covariate values. In the future, we anticipate that the results of our study will contribute to predicting IOH in real time in the operating room and reducing the occurrence of IOH.

## Figures and Tables

**Table 1:** Dataset characteristics.

Characteristic	Total (N = 2611)	Train (N = 2088)	Test (N = 523)
Age (years)	59.8 ± 14.0	59.7 ± 14.0	60.0 ± 14.3
Male, n (%)	1451 (55.6%)	1166 (55.8%)	285 (54.5%)
BMI (kg/m^2^)	23.1 ± 3.5	23.1 ± 3.5	23.3 ± 3.6
ASA physical status			
I	552 (21.1%)	440 (21.1%)	112 (21.4%)
II	1686 (64.6%)	1353 (64.8%)	333 (63.7%)
III	344 (13.2%)	275 (13.2%)	69 (13.2%)
IV	18 (0.7%)	13 (0.6%)	5 (1.0%)
V	0	0	0
VI	11 (0.4%)	7 (0.3%)	4 (0.8%)
Emergency surgery	288 (11.0%)	231 (11.1%)	57 (10.9%)
Hypertension	856 (32.8%)	699 (33.5%)	157 (30.0%)
WBC count (10^3^/mcL)	5.8 ± 2.1	5.8 ± 2.1	5.8 ± 2.1
Hemoglobin (g/dL)	10.7 ± 2.0	10.7 ± 2.0	10.8 ± 2.1
BUN (mg/dL)	9.7 ± 4.8	9.7 ± 4.9	9.7 ± 4.3
Cr (mg/dL)	0.7 ± 0.6	0.7 ± 0.6	0.7 ± 0.6
Albumin (g/dL)	3.1 ± 0.6	3.1 ± 0.6	3.2 ± 0.6
Na (mmol/L)	133.5 ± 3.3	133.5 ± 3.2	133.5 ± 3.4
K (mmol/L)	3.4 ± 0.4	3.4 ± 0.4	3.4 ± 0.4

BMI, Body mass index; ASA, the American Society of Anesthesiologists; WBC, White blood cell; BUN, Blood urea nitrogen; Cr, Creatinine; Na, Sodium; K, Potassium.

**Table 2:** Performance of the machine learning and deep learning models.

Metric	GBM	Hybrid CNN-RNN	*p*-Value	Test
AUROC (95% CI)	0.94 (0.93–0.95)	0.94 (0.93–0.95)	<0.001	DeLong
Accuracy (95% CI)	0.88 (0.86–0.89)	0.88 (0.87–0.89)	0.591	McNemar
Sensitivity (95% CI)	0.83 (0.81–0.85)	0.80 (0.78–0.82)	<0.001	McNemar (positive subset)
Specificity (95% CI)	0.90 (0.89–0.92)	0.93 (0.92–0.94)	<0.001	McNemar (negative subset)
PPV (95% CI)	0.84 (0.83–0.86)	0.88 (0.86, 0.89)	<0.001	Two-proportion (*z*-test)
NPV (95% CI)	0.89 (0.88–0.91)	0.88 (0.87, 0.89)	0.003	Two-proportion (*z*-test)

AUROC, area under the receiver operating characteristic curve; GBM, gradient boosting machine; CNN, convolutional neural network; RNN, recurrent neural network; PPV, positive predictive value; NPV, negative predictive value.

## Data Availability

These data were derived from the following resources available in the public domain: [https://vitaldb.net/] (accessed on 1 March 2023).
